# TIM-1 Promotes Japanese Encephalitis Virus Entry and Infection

**DOI:** 10.3390/v10110630

**Published:** 2018-11-14

**Authors:** Jichen Niu, Ya Jiang, Hao Xu, Changjing Zhao, Guodong Zhou, Puyan Chen, Ruibing Cao

**Affiliations:** MOE Joint International Research Laboratory of Animal Health and Food Safety, College of Veterinary Medicine, Nanjing Agricultural University, Nanjing 210095, China; 2016107039@njau.edu.cn (J.N.); 2015107042@njau.edu.cn (Y.J.); 2016807118@njau.edu.cn (H.X.); 2015107041@njau.edu.cn (C.Z.); zgd@njau.edu.cn (G.Z.); puyanchennj@163.com (P.C.)

**Keywords:** Japanese encephalitis virus, TIM-1, polymorphism, infection, entry cofactor

## Abstract

Japanese encephalitis virus (JEV) is a mosquito-borne *Flavivirus*, the leading cause of viral-induced encephalitis. Several host molecules have been identified as the JEV attachment factor; however, the molecules involved in JEV entry remain poorly understood. In the present study, we demonstrate that TIM-1 is important for efficient infection by JEV. Firstly, three TIM-1 variants (V1, V2, and V3) were cloned from A549 cells, and we revealed that only ectopically TIM-1 V2 expression in 293T cells significantly promotes JEV attachment, entry and infection. Point mutation of phosphatidylserine (Ptdser) binding pocket in the TIM-1 IgV domain dampened JEV entry, indicating that TIM-1-mediated JEV infection is Ptdser-dependent. Furthermore, we found the cytoplasmic domain of TIM-1 is also required for enhancing JEV entry. Additionally, knock down of TIM-1 expression in A549 cells impaired JEV entry and infection, but not attachment, suggesting that additional factors exist in A549 cells that allow the virus to bind. In conclusion, our findings demonstrate that TIM-1 promotes JEV infection as an entry cofactor, and the polymorphism of TIM-1 is associated with JEV susceptibility to host cells.

## 1. Introduction

Japanese encephalitis (JE) is a severe epidemic of viral encephalitis with a high fatality rate, and it is caused by the Japanese encephalitis virus (JEV) [1]. In eastern Asia, JEV infections cause about 30,000 to 50,000 cases of encephalitis that lead to 10,000 deaths annually. Currently, a specific treatment for JE is lacking, and survivors usually develop significant neurological sequelae [2]. JEV is an enveloped virus containing a single strand of positive-sense RNA and it belongs to the *Flavivirus* genus. JEV is mosquito-borne, and it maintains a zoonotic cycle between wading birds or pigs and Culex mosquitoes [1,3]. The genomic RNA of JEV is approximately 11 knt. It encodes a large precursor polyprotein, which is cleaved into three structural proteins and seven nonstructural proteins by host and virus-encoded proteases. The envelope E glycoprotein is considered a major antigenic determinant that mediates cellular attachment and membrane fusion [4].

JEV infects various types of cell from a wide range of mammalian, avian, amphibian, and insect species, which indicates that JEV can utilize multiple receptors to enter different cells [5]. The current study suggests that *Flaviviruses* use two different types of molecules for infection, an attachment factor that recruits viral particles on the cell membrane and primary receptors that bind to virions and initiate the endocytic pathway [6]. *Flaviviruses* interact with several low-affinity attachment factors, including heparin-sulfate proteoglycans and low-density lipoprotein receptors [7,8,9]. Previous studies have proposed multiple cellular molecules as JEV candidate receptors for different cell types. The C-type lectin receptor DC-specific ICAM-3 grabbing nonintegrin (DC-SIGN) has been proposed as an attachment factor that mediates JEV infection in dendritic cells (DCs) by interacting with E glycoprotein [10]. Integrin αVβ3 was also demonstrated as the primary receptor for JEV [11]. However, integrin αVβ3 is not essential for viral entry in certain cellular contexts [12]. Other studies have suggested that HSP70 is a putative receptor for JEV in murine neuroblastoma cells [13]. A recent study has shown that HSP90β binds JEV to the membranes of Vero cells, which indicates that HSP90β serves as a JEV receptor in Vero cells [14]. In summary, JEV infection is a complex process and, currently, studies identifying the functional receptors utilized by JEV to infect a diverse set of susceptible cells remain elusive.

T-cell immunoglobulin and mucin domain 1(TIM-1) is a type I transmembrane glycoprotein with an extracellular domain composed of an N-terminal immunoglobulin V (IgV)-like domain followed by a glycosylated mucin domain, a transmembrane domain, and a short cytoplasmic tail [15]. TIM-1 has been reported to enhance various RNA virus infections, including Dengue virus (DENV), West Nile virus (WNV), Zika virus (ZIKV), Ebola virus (EBOV), Marburg virus (MARV), hepatitis A virus (HAV) and hepatitis C virus (HCV) [16,17,18,19,20,21,22], and it is thought to be an important regulator of immune tolerance [23]. TIM-1-mediated enhancement of infection by enveloped viruses is mainly dependent on the association of Ptdser with viral particles. TIM-1 can directly bind to Ptdser exposed on the viral envelope. The natural function of TIM-1 is to interact with Ptdser and modulate phagocytosis. The viruses hijack this process for entry, which is called the “apoptotic mimicry” strategy [24,25,26,27,28]. The Ptdser-binding site is a pocket located between CC′ and FG loops. It is a conserved cavity within the TIM-1 IgV-domain that is called the metal ion-dependent ligand-binding site (MILIBS) [18]. In addition to functioning as a Ptdser receptor, TIM-1 acts as a dual receptor by directly interacting with the glycoprotein of Ebola virus and Ptdser exposed on the surface of the viral envelope [29]. Moreover, alternative splicing produces different forms of TIM-1 that are identical, except for the C-terminal portions of the cytoplasmic domains. The tissue-specific distributions of these isoforms indicate distinct roles for TIM-1 in different tissues [30]. The human TIM-1 gene has three haplotypes in the mucin domain: a short form, an intermediate form with a 5-amino acid insertion (157ins MTTVP), and a long form with a 6-amino acid (157ins MTTTVP) insertion. Previous studies have reported that the 6-amino acid insertion (157ins MTTTVP) was associated with several severe diseases [31]. Polymorphisms of TIM-1 are associated with cell susceptibility to infection by several viruses, including HIV and HAV [21,32,33]. However, the role of TIM-1 in JEV infection and if TIM-1 polymorphisms are involved in cells susceptibility to JEV is still unknown.

In this study, we carried out a series of experiments that ectopically express TIM-1 in 293T cells and silence endogenous TIM-1 expression in A549 cells using RNAi technology to investigate the role of TIM-1 in JEV infection. Our results suggest that TIM-1 significantly promotes JEV infection as an entry cofactor. Furthermore, we show that the polymorphism of TIM-1 was associated with JEV susceptibility to host cells.

## 2. Materials and Methods

### 2.1. Cells and Virus Preparation

Baby hamster kidney (BHK-21) cells and human embryonic kidney 293T (HEK-293T) cells were grown in Dulbecco’s modified essential medium (DMEM, GIBCO, Invitrogen, Carlsbad, CA, USA) supplemented with 10% fetal bovine serum (FBS) (GIBCO), 100 U/mL penicillin–streptomycin, at 37 °C in 5% CO_2_. A549 cells and C6/36 cells were cultured in RPMI-1640 (GIBCO) supplemented with 10% FBS, 100 U/mL penicillin–streptomycin, at 37 °C and 28 °C, respectively, in 5% CO_2_. The JEV NJ2008 (GenBank: GQ918133) and HN07 strains were maintained in our laboratory and propagated in C6/36 cell. Virus titers were assessed by a plaque assay titrated on BHK-21 cells. The live-attenuated Japanese encephalitis vaccine virus SA14-14-2 strain was purchased from Wuhan Keqian Biology Co., Ltd. (Wuhan, China); they were grown and titrated on BHK-21 cells.

### 2.2. Virus Challenge and Titration

Virus infection and titration were performed as previously described [34]. Briefly, 293T cells were plated on poly-lysine-coated (plates were pretreated with poly-lysine at 37 °C for 30 min), other cells were seeded in uncoated plates, incubated with indicated multiplicity of infection (MOI) of JEV strain NJ2008, HN07, or SA14-14-2 at 37 °C for 1 h. They were then washed to remove unbound virions and cultured with fresh medium at 37 °C for the indicated times. The supernatants of cells were collected, and virus titers were determined by a plaque-forming assay titered on monolayer BHK-21 cells.

### 2.3. Plasmid Construction and Antibodies

Total RNA was extracted from A549 cells by Trizol (TaKaRa Bio, Kyoto, Japan), and then it was reverse transcribed to produce cDNA using PrimeScript™ II 1st Strand cDNA Synthesis Kit (TaKaRa). The full-length sequence of the gene encoding TIM-1 was amplified by polymerase chain reaction (PCR) from A549 cells. The TIM-1 gene open reading frame (ORF) was cloned into pcDNA3.1 (−) using *BamH*I and *Xba*I restriction sites. The ORF of TIM-1 was amplified with oligos 5′-GCTCTAGAATGCATCCTCAAGTGG-3′ and 5′-ATGGATCCTTAGTCCGTGGC-3′. Specific monoclonal antibodies for JEV E and NS1 were produced in our lab, and an NS5 monoclonal antibody was purchased from Gene Tex (Irvine, CA, USA). A GAPDH polyclonal antibody (sc-25778) was purchased from SantaCruz (Dallas, TX, USA). A TIM-1 monoclonal antibody (MAB1750) and polyclonal antibody (AF1750) were purchased from R&D systems.

### 2.4. RNA Interference and Plasmid Transfection

A549 cells were seeded in 12-well or 24-well plates overnight, and they were transfected with indicated concentration of siRNA using Lipofectamine RNAiMAX Reagent. At 48 h post-transfection, cells were infected with JEV at the indicated MOI. Pools of TIM-1 siRNA (sc-61691) and control siRNA (sc-37007) were purchased from SantaCruz. Plasmid transfection was performed using Lipofectamine 3000 according to manufacturer’s instructions. At 24 h post-transfection, cells were infected with JEV at the indicated MOI. JEV infection was determined at different time point by Western blot analysis, quantitative reverse transcription PCR (qRT-PCR), and plaque assay.

### 2.5. Virus Attachment and Entry Assays

Cells were seeded in 24-well poly-lysine coated (293T) or uncoated (A549) cell culture plates overnight. Then cells were transfected with indicated plasmids or siRNAs for 24 h (ectopic expression) or 48 h (knockdown). Cells were incubated with JEV of indicated MOI at 4 °C for 30 min (attachment assay). Unbound JEV virions were removed by washing with phosphate-buffered saline (PBS) three times. Total RNA was extracted and used for quantification of JEV RNA by a qRT-PCR method. JEV entry assays was carried out by incubation of JEV with cells at 4 °C for 1 h to allow JEV binding, and then unbound JEV virions were washed three times with PBS rapidly. Then cells were shifted to 37 °C for 15 min to allow JEV internalization, and then treated with proteinase K (1 mg/mL) to remove non-internalized virions, as described previously [35].

### 2.6. Real-Time Polymerase Chain Reaction (PCR)

The total RNA in JEV-infected cells was extracted by the Total RNA extract KIT (OMEGA, Bio-tek, Norcross, GA, USA), and cDNA was synthesized from 500 ng RNA using the HiScript ® II 1st Strand cDNA Synthesis Kit (+gDNA wiper) (Vazyme, Nanjing, China). The level of JEV RNA was determined by qRT-PCR, using TB Green Premix Ex Taq (TliRNaseH Plus) (Takara) and specific primers and probes in the 7300 real-time PCR system (Applied Biosystems, Foster City, CA, USA). The cellular GAPDH mRNA was used as an internal control. The primers for JEV RNA, TIM-1 mRNA, and GAPDH mRNA quantification were as follows: 1aF (5′-GAGTCAACGGATTTGGTCGT-3′) and 1aR (5′-GACAAGCTTCCCGTTCTCAG-3′) are complementary to GAPDH sequence; primers for JEV E 2aF (5′-GGCAAACGACAAACCAACATT-3′) and 2aR (5′-ATCAGCTCGCTTCTCGTTGTG-3′); primers for TIM-1 3aF (5′-AACTGTCTCTACCTTTGTTCCTCC-3′) and 3aR (5′-GTTCTCTCCTTATTGCTCCCTG-3′). The quantification of the relative expression was carried out based on the comparative CT Method, using GAPDH as an endogenous reference control.

### 2.7. Western Blot Analysis

Cells were washed with PBS three times, and then lysed in radioimmunoprecipitation assay (RIPA) buffer (Pierce, Thermo Fisher, Cambridge, MA, USA) for 15 min. Then, the supernatants were collected after centrifugation, and the concentrations of protein were measured by BCA Protein Assays (Pierce). Then, 40 µg of total protein from each sample was separated by 10% sodium dodecyl sulphate-polyacrylamide gel electrophoresis (SDS-PAGE). Proteins were transferred to a polyvinylidene difluoride (PVDF) membrane (Bio-RAD Laboratories, Hercules, CA, USA). Following blocking with 5% nonfat milk, the membranes were incubated with their indicated primary antibody overnight. After washing three times using PBST, the membrane was incubated with goat anti-mouse or goat anti-rabbit horseradish peroxidase (HRP)-conjugated IgG antibody (Pierce). The BIO-RAD Clarity Western ECL Substrate was used to detect the protein levels.

### 2.8. Confocal Microscopy

Cells were cultured on Glass Bottom Culture Dishes (NEST, Wuxi, China) overnight and transfected with indicated plasmid. At 24 h post-transfection, cells were infected with JEV (NJ2008) at the indicated MOI. At different times point, cells were fixed with 4% PBS-paraformaldehyde (PFA) and permeabilized with 0.1% Triton X-100. They were then incubated with a primary antibody and washed three times with PBS. Then, they were stained with a secondary antibody of donkey anti-goat Alexa Fluor488 (ab150129) (Abcam, Cambridge, UK) and donkey anti-mouse Alexa Flour647 (ab150107) (Abcam). Nuclei staining were accomplished using 4,6-diamidino-2-phenylindole (DAPI).

### 2.9. Statistical Analysis

Graphical and statistical analyses were performed using GraphPad Prism6.0 software (GraphPad Software Inc., La Jolla, CA, USA). Values are given as the mean of triplicates with standard deviation (SD). Statistical analysis was performed using an unpaired two-tailed t-test. A *p* value < 0.05 was considered statistically significant.

## 3. Results

### 3.1. 293T Cells Are Poorly Permissive to JEV Infection at Early Stages

**Figure 1:**
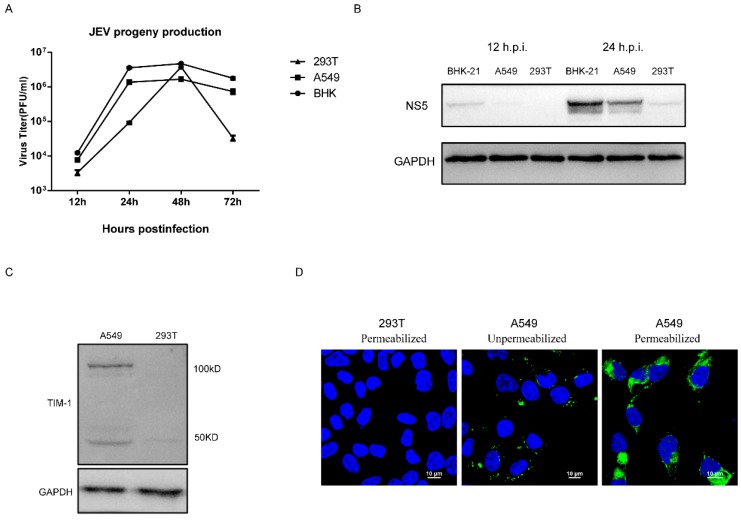
JEV infection and TIM-1 expression in cell lines. BHK, A549, and 293T cells were infected with JEV NJ2008 (MOI of 0.5) for 40 h. (**A**) Supernatants were collected and the production of progeny virions were determined by a plaque assay titrated on BHK-21 cells. Data are presented as mean ± standard deviation (SD) from three independent experiments; (**B**) Cell lysates were harvested to detect the level of JEV NS5 and TIM-1 protein by Western blot analysis using specific antibodies. One representative experiment out of three is shown. Endogenous TIM-1 expression in A549 and 293T cells was detected by (**C**) Western blot analysis and (**D**) confocal microscopy using an anti-TIM-1 monoclonal antibody. Scale bars, 10 µm.

JEV can infect various cell lines, so it is difficult to find a cell line that is non-permissive to JEV. The 293T cell is reported to be non-permissive to DENV, which is a *Flavivirus* closely related to JEV. Therefore, we examined whether 293T cell is comparatively poorly permissive to JEV, and we compared the susceptibility of three cell lines to JEV. 293T, A549, and BHK-21 cells were infected with JEV NJ2008 at a multiplicity of infection (MOI) of 0.5, cell lysates and supernatants were harvested at different times post-infection. The JEV infection efficiency was determined by Western blot analysis using an NS5 monoclonal antibody. Virus titers in the supernatants of infected 293T, A549, and BHK cells at 24 h.p.i. were approximately 1.0 × 10^5^ PFU/mL, 1.4 × 10^6^ PFU/mL, and 3.6 × 10^6^ PFU/mL, respectively (Figure 1A). The production of JEV progeny in A549 and BHK cells was increased 14-fold and 36-fold, respectively, compared to 293T cells at 24 h.p.i. JEV progeny production in A549 and BHK cells were increased 2-fold and 4-fold, respectively, at 12 h.p.i. We observed more virus production in A549 and BHK cells than 293T cells before 48 h post infection. Western blot analysis revealed that the level of JEV NS5 protein in infected 293T cells was nearly undetectable at 12 h post-infection (h.p.i.), and it was low at 24 h.p.i. The level of JEV NS5 protein in A549 cells and BHK cells were much more than that in 293T cells at 24 h.p.i. (Figure 1B). This indicates that 293T cells are permissive to JEV, but they were poorly permissive at the early stage of JEV infection. Consistent with previous reports [36], Western blot analysis confirmed that 293T cells express a low level of TIM-1. Furthermore, we observed the expression of two specific bands of TIM-1 in A549 cells. The molecular weights of the two bands were about 50 kDa and 100 kDa, which indicates that TIM-1 exists in different forms in host cells (Figure 1C). Using confocal microscopy, we observed abundant TIM-1 in A549 cells, as indicated by the green spots under unpermeabilized condition. We could see that TIM-1 was highly expressed both in cytoplasm and on cell membrane under permeabilized condition, but no obvious TIM-1 signals were detected in 293T cells (Figure 1D). Therefore, 293T cells were used as a cell model for subsequent experiments because they are less permissive to JEV, and lack endogenous TIM-1 expression.

### 3.2. Human TIM-1 Gene Clone and Polymorphism Analysis

**Figure 2:**
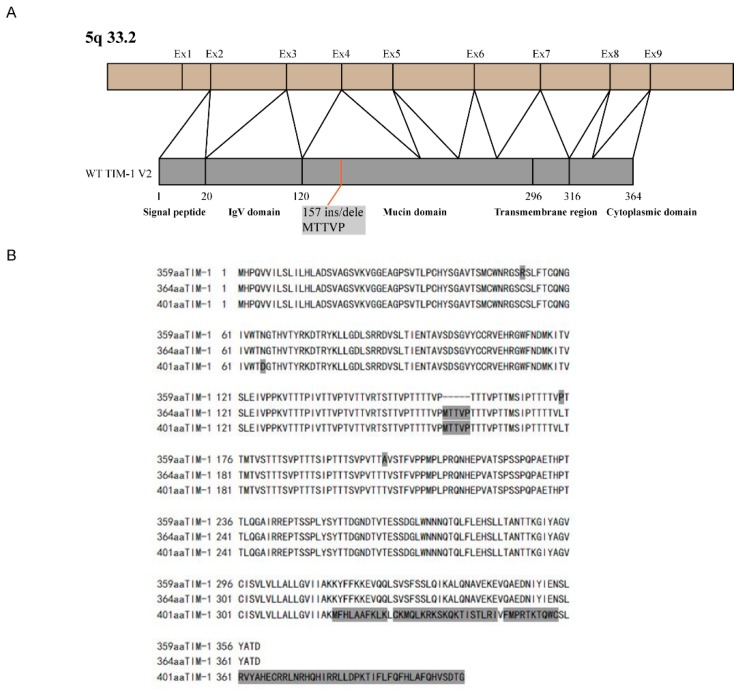
Human TIM-1 amino acid sequence and structural domains. (**A**) TIM-1 gene localization, and construction of TIM-1 protein functional domain; (**B**) amino acid sequence of the three TIM-1 variants with their differences indicated in gray.

TIM-1 was the first TIM protein identified as a cellular receptor for HAV (HAVcr-1) in humans and African green monkeys. It is also associated with allergy and asthma diseases. The human *TIM-1* gene is located at chromosome 5q33.2. It is highly polymorphic, primarily in exon 4, which encodes the mucin domain (Figure 2A). To date, a large number of genetic studies have been carried out to investigate a possible association of various TIM-1 polymorphisms with different diseases [37,38]. TIM-1 is broadly expressed in many cell lines, including A549 cells, and it is involved in mediating a wide range of viral infections [19]. Thus, we extracted total RNA from A549 cells, and cloned three human *TIM-1* gene variants using RT-PCR, *401aa TIM-1* (TIM-1 V1), *364aa TIM-1* (TIM-1 V2), and *359aa TIM-1* (TIM-1 V3). TIM-1 V1 has an N65D mutation, as compared with *401aa TIM-1* (Accession: NP_001295085.1). TIM-1 V2 contains the 5-amino acid insertion 157ins MTTVP (Accession: NP_036338.2). TIM-1 V3 has the mutations of C52R and T202A, as compared with *359aa TIM-1* (GenBank: AFO66592.1). The amino acid sequence differences among the three variants are shown in Figure 2B.

### 3.3. Ectopic TIM-1 V2 (364aa) Expression in 293T Cells Markedly Enhances JEV Infection

**Figure 3:**
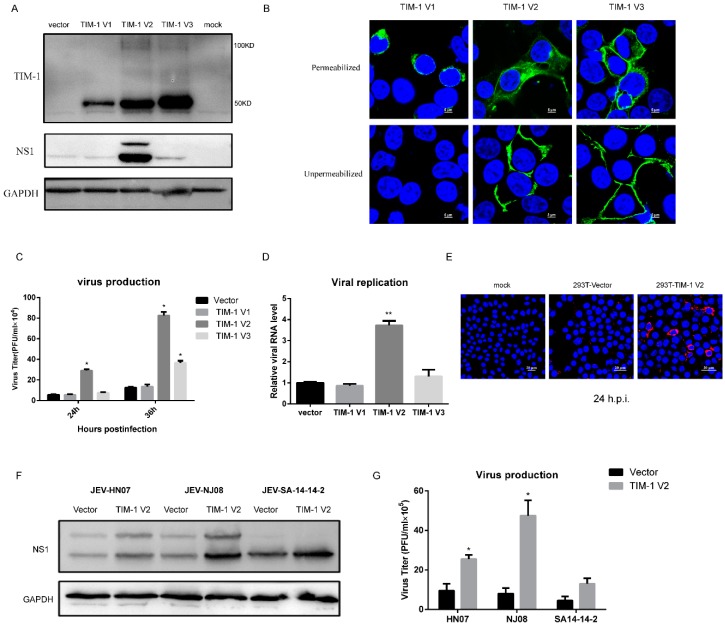
Polymorphisms of TIM-1 are associated with JEV susceptibility to host cells. 293T cells were transfected with an empty vector and plasmids encoding three *TIM-1* variants for 24 h. (**A**) Cells were challenged with JEV NJ2008 (MOI of 1), and cell lysates were harvest at 24 h.p.i. JEV NS1 protein and TIM-1 expression were determined by Western blot analysis using a specific monoclonal antibody. One representative experiment out of three is shown; (**B**) cells were fixed and detected by confocal microscopy under permeabilized and unpermeabilized condition. Scale bars, 5 µm; (**C**) supernatants were collected at 24 h.p.i. and 36 h.p.i. and the production of progeny virions was determined by a plaque assay titered on BHK-21 cells. Data are presented as mean ± SD from three independent experiments using t-test. * *p* < 0.05; (**D**) cells were transfected with indicated plasmids for 24 h and infected with JEV NJ2008 (MOI of 1). Total RNA was extracted and viral replication was detected by qRT-PCR at 24 h.p.i. Data are presented as mean ± SD from three independent experiments using *t*-test. ** *p* < 0.01; (**E**) 293T cells were transfected with indicated plasmids for 24 h. Cells were incubated with JEV (MOI = 1) for 24 h. Then they were fixed and stained with anti-E monoclonal antibody. Scale bars, 20 µm. 293T cells transfected with a plasmid encoding TIM-1 V2 or an empty vector were infected with different strains of JEV (HN07, NJ2008, and SA14-14-2) at a 0.5 MOI; (**F**) cell lysates were harvested at 40 h.p.i. and JEV infection was determined by the level of NS1 protein using Western blot analysis. One representative experiment out of three is shown; (**G**) supernatants were collected and the production of JEV virions were determined by a plaque assay titrated on BHK-21 cells.

Previous studies have reported that TIM-1 promotes infection of many enveloped viruses, including several members of the Flaviviridae family [39]. To examine the distinct roles of three TIM-1 variants during JEV infection, we carried out gain-of-function experiments by ectopic TIM-1 expression in 293T cells. 293T cells were transfected with an empty plasmid or a TIM-1-encoding plasmid, and then they were inoculated with JEV NJ2008. Then, supernatants and cell lysates were harvested at different times post-infection. Our data show that, compared with control cells, there was no significant change in NS1 protein levels of JEV infected 293T cells transiently expressing TIM-1 V1 and V3. Interestingly, JEV NS1 protein levels has a remarkably increase in cells expressing TIM-1 V2 (Figure 3A). Moreover, TIM-1 V2 and V3 had two forms of TIM-1 in 293T cells, with molecular weights of about 50 kDa and 100 kDa, respectively. However, only the 50 kDa TIM-1 was found in 293T cells expressing TIM-1 V1. Moreover, the predicted molecular mass of TIM-1 V3 is smaller than that of TIM-1 V2, but the TIM-1 V3 protein band is at a higher molecular mass. This indicates that the modification of TIM-1 V2 and V3 is different. Previous studies have revealed that the 100 kDa TIM-1 is the form distributed on the cell membrane [30]. The predicted molecular mass of TIM-1 is 36 kDa, but there are four potential N-glycosylation sites and multiple O-glycosylation sites in the mucin domain, which would result in a higher protein molecular mass. In order to confirm the distribution of three variants in cells, we performed a confocal microscopy assay to observe the distribution of TIM-1 V1, V2 and V3 in cells under permeabilized and unpermeabilized condition (Figure 3B). Our results show that TIM-1 V1 distributes in the cytoplasm but not on the cell surface, and appropriate glycosylation could be important for TIM-1 to enhance JEV infection. The viral titer of supernatants from JEV-infected cells transiently expressing TIM-1 V2 and V3 show that the production of JEV progeny was about 6-fold and 1.6-fold higher, respectively, than 293T cells transfected with the empty vector at 24 h.p.i. (Figure 3C). The same trend was also observed at 36 h.p.i. As expected, the replication of JEV in 293T cells transiently expressing TIM-1 V1, V2, and V3 was approximately 0.8-fold, 3.7-fold, and 1.3-fold, respectively, compared to control 293T cells (Figure 3D). As a result, the ectopic expression of TIM-1 V1 in 293T cells had no significant effects on JEV infection, which is indicated by a similar level of infection efficiency to control cells. We observed a significant increase in JEV infection (as indicated by red spots) in 293T cells transiently expressing TIM-1 V2, when compared to 293T cells control at 24 h.p.i. (Figure 3E). These results further confirm that TIM-1 V2 promotes JEV infection. Cells transfected with an empty vector or a plasmid encoding TIM-1 V2 were infected with three strains of JEV (JEV I HN07, JEV III NJ2008, and live-attenuated Japanese encephalitis vaccine virus SA14-14-2) at an MOI of 0.5 for 40 h. We observed that ectopic TIM-1 V2 expression in 293T cells increased the NS1 protein levels of three different strains of JEV (SA14-14-2 is a vaccine virus strain, which do not produce NS1’ protein) (Figure 3F). A virus titer in TIM-1 V2 expressing cells was approximately 2.7-fold, 5.9-fold and 3.2-fold as control cells, respectively (Figure 3G). Gain-of-function studies showed that TIM-1 V2 expression increased the susceptibility of 293T cells to all the three JEV strains. Taken together, our results indicate that TIM-1 V2 markedly enhances JEV infection in 293T cells, and we identify that polymorphisms of TIM-1 involves in JEV susceptibility to target cells.

### 3.4. TIM-1 Promotes JEV Attachment and Internalization, and May Be Associated with the Intracellular Trafficking of Virions

**Figure 4:**
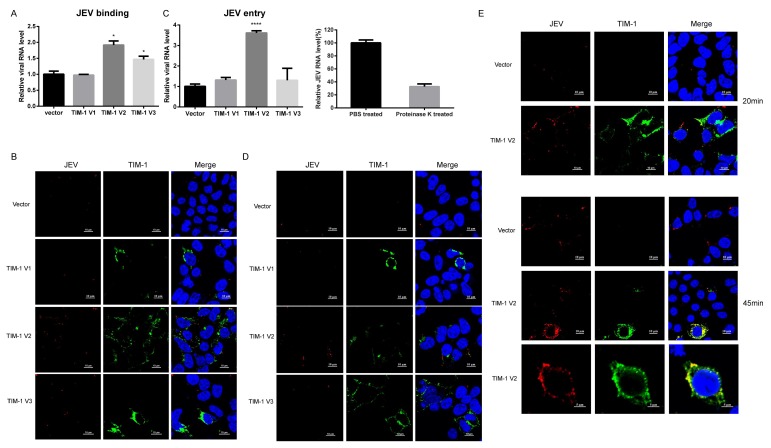
TIM-1 V2 enhances JEV attachment and entry. 293T cells were transfected with an empty vector or a plasmid encoding TIM-1 variant for 24 h. (**A**) They were incubated with JEV (MOI = 5) at 4 °C for 30 min, and washed with PBS three times. Total RNA was extracted and used for quantification of JEV RNA by qRT-PCR. Data are presented as mean ± SD from three independent experiments using t-test. * *p* < 0.05; (**B**) cells were incubated with JEV NJ2008 (MOI of 20) at 4 °C for 30 min and fixed followed by confocal microscopy. Scale bars, 10 µm; (**C**) cells were challenged with JEV NJ2008 (MOI = 5) at 4 °C for 1 h. They were then washed with PBS (or treated with proteinase K to examine the efficiency of removing JEV virions on the cell membrane) to remove unbound JEV particles and then transferred to 37 °C for 15 min to allow virus entry. Total RNA was extracted and used for quantification of JEV RNA by qRT-PCR. Data are presented as mean ± SD from three independent experiments using t-test. **** *p* < 0.0001; (**D**) cells were incubated with JEV NJ2008 (MOI of 10) at 4 °C for 1 h. Then, they were transferred to 37 °C for 15 min, treated with proteinase K (1 mg/mL) and fixed with 4% PBS-PFA. They were stained with anti-E monoclonal antibody and anti-TIM-1 polyclonal antibody. Scale bars, 10 µm; (**E**) cells were transfected with the indicated plasmids for 24 h, and then incubated with JEV NJ2008 (MOI of 10) at 37 °C for 20 min and 45 min. Cells were fixed and detected by confocal microscopy. Scale bars, 10 µm or 5 µm.

Since it is a class I transmembrane protein, we examined the function of TIM-1 during JEV binding and internalization. Cells were incubated with JEV NJ2008 at 4 °C for 30 min to allow virus binding. Then total RNA was extracted, and the efficiency of JEV binding was detected by qRT-PCR. We observed that TIM-1 V2 and V3 significantly enhanced the binding of JEV particles to 293T cells. TIM-1 V2 and V3 expression increased JEV binding efficiency to approximately 1.9-fold and 1.5-fold, respectively, compared to 293T control cells. However, TIM-1 V1 expression had almost no effect on JEV binding to target cells (Figure 4A). Using confocal microscopy, we observed a robust increase in JEV on the surface of cells expressing TIM-1-V2. We also found that TIM-1 V2 colocalized with JEV, which indicates that TIM-1 mediates the binding of JEV to cells (Figure 4B). Following attachment, endocytosis is the second step during early virus infection, so we carried out an internalization assay to investigate if TIM-1 plays a role in JEV cellular entry. Cells were incubated with JEV NJ2008 at 4 °C for 1 h to allow viral particle binding, then washed extensively with cold PBS (or treated with proteinase K to examine the efficiency of removing JEV virions on the cell membrane). Following incubation at 37 °C to allow internalization of viral particles, cells were treated with proteinase K to remove the cell-surface bound JEV virions, as previously described [40]. Total RNA was extracted, and the efficiency of JEV entry was evaluated by qRT-PCR. The results show that the efficiency of JEV entry into 293T cells transiently expressing TIM-1 V1, V2, and V3 was increased 1.3-fold, 3.6-fold, and 1.3-fold, respectively, compared to 293T cells transfected with an empty vector (Figure 4C). After proteinase K treated, our data show about 68% JEV virions on cell membrane were removed. We then performed fluorescence confocal microscopy to observe the process of JEV entry. We observed there was more JEV entry into cells expressing TIM-1 V2 compared to control cells. However, there was no difference between the number of JEV entering cells expressing TIM-1 V1 and V3 (Figure 4D). Furthermore, we observed that TIM-1 V1 distributed around the nuclei and was not located not on the cell membrane, which is consistent with our previous findings. To examine the early stage of the process of JEV infection, we made observations at two different time points during JEV entry into cells (20 min and 45 min). There were more JEV entering the cells expressing TIM-1 V2 at 20 min post-inoculation, compared to control cells, and a portion of the green spots and red spots overlapped. These results indicate that TIM-1 colocalizes with JEV. At 45 min post-infection, we observed that most JEV gathered around the cells expressing TIM-1 V2. Numerous JEV colocalized with TIM-1, and the JEV were found closer to nuclei (Figure 4E). Taken together, our data suggest that TIM-1 V2 promotes JEV attachment and internalization, and may be involved in intracellular trafficking after JEV enters host cells.

### 3.5. Phosphatidylserine-Binding Capability of TIM-1 is Crucial for Mediating JEV Attachment, Entry, and Infection

**Figure 5:**
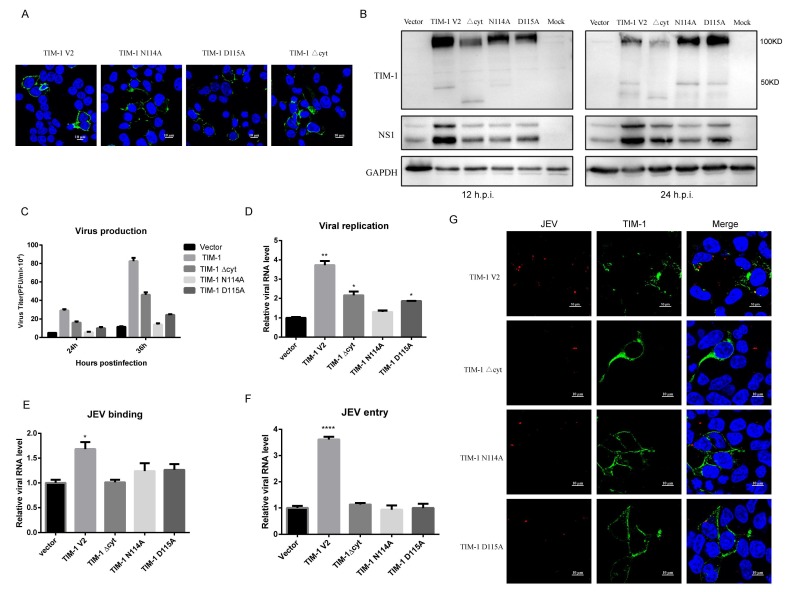
TIM-1 mutants were unable to promote JEV infection. (**A**) Localization of WT TIM-1 and TIM-1 mutants in 293T cells were examined by confocal microscopy. Scale bars, 10 µm. 293T cells were transfected with WT TIM-1 or TIM-1 mutants encoding plasmids for 24 h, and then cells were challenged with JEV NJ2008 of different MOI; (**B**) cell lysates were collected at 12 h.p.i. (MOI = 5) and 24 h.p.i. (MOI = 1), and JEV infection was determined by Western blot analysis using an NS1-specific monoclonal antibody. One representative experiment out of three is shown; (**C**) supernatants were collected at 24 h.p.i. and 36 h.p.i. JEV progeny production was detected by plaque assay. Data are presented as mean ± SD from three independent experiments; (**D**) total RNA was extracted at 24 h.p.i. and viral replication was determined by qRT-PCR. Data are presented as mean ± SD from three independent experiments using t-test. * *p* < 0.05, ** *p* < 0.01; (**E**) cells were incubated with JEV NJ2008 (MOI of 5) at 4 °C for 30 min and then washed with PBS three times. Total RNA was extracted, and the efficiency of JEV attachment was quantified by qRT-PCR. Data are presented as mean ± SD from three independent experiments using t-test. * *p* < 0.05; (**F**) cells were incubated with JEV NJ2008 (MOI of 5) at 4 °C for 1 h, and then they were washed three times with PBS. They were then shifted to 37 °C for 15 min and treated with proteinase K (1 mg/mL). Total RNA was extracted, and the efficiency of JEV entry was quantified by qRT-PCR. Data are presented as mean ± SD from three independent experiments using t-test. **** *p* < 0.0001; (**G**) cells transiently expressing TIM-1 and the mutants were challenged with JEV NJ2008 (MOI = 10) for 1 h at 4 °C. They were then shifted to 37 °C for 15 min. Cells were treated with proteinase K (1 mg/mL). Then, they were fixed and stained for E glycoprotein (red) and TIM-1 (green), followed by confocal microscopy analysis. Scale bars, 10 µm.

To explore the possible mechanisms of TIM-1 mediated JEV infection, we generated two mutants of highly conserved amino acids (N114A, D115A). The N114 and D115 amino acids are found in the Ptdser binding site and are crucial to Ptdser recognition. We firstly confirmed the localization of WT TIM-1 and TIM-1 mutants expressed in 293T cells using fluorescence confocal microscopy. We found that WT TIM-1 and TIM-1 mutants (N114A/D115A) localize on the cell membrane, and the expression levels of the two mutants were similar to that of WT TIM-1 (Figure 5A,B). Additionally, mutants resulted in a lower level of cellular JEV NS1 protein compared to WT TIM-1. Compared to WT TIM-1, the N114A and D115A mutants impared the ability to mediate JEV infection, but still enhance JEV infection (Figure 5B). Similarly, at 24 h.p.i, there were ~83% and ~70% decreases in JEV production in 293T cells transiently expressing the N114A and D115A TIM-1 mutants, respectively (Figure 5C). There was also a similar trend observed at 36 h.p.i. Our data show that, compared to control cells, the genomic RNA of JEV was increased to 3.7-fold in WT TIM-1, 1.3-fold in N114A TIM-1, and 1.9-fold in D115A TIM-1 at 24 h.p.i. (Figure 5D). These results suggest that mutations at N114 and D115 markedly impair the function of TIM-1 in mediating JEV infection. Subsequently, we performed attachment and internalization assays (as previously described [35] to assess the effects of the two TIM-1 mutants on JEV binding and entry. According to our results, WT TIM-1, N114A and D115A mutants expression in 293T cells increased the efficiency of JEV binding by 1.7-fold, 1.2-fold, and 1.2-fold, respectively, compared to 293T control cells. These data suggest that the N114A and D115A mutations of TIM-1 dampened the function of mediating JEV binding to cells (Figure 5E). Moreover, ectopic expression of the TIM-1 N114A and D115A mutants had a similar efficiency of JEV entry as that of cells transfected with an empty vector (Figure 5F). This suggests that these mutations can also halt the ability of TIM-1 to mediate the internalization of JEV. To further observe the process in which JEV enters cells, we performed confocal microscopy assay. There were markedly more JEV entry into cells expressing TIM-1 V2, and there were considerably less JEV entering cells expressing TIM-1 mutants. We also found there were no colocalizations between JEV and TIM-1 mutants (Figure 5G). Taken together, our data demonstrate that the PS-binding pocket of TIM-1 is important for JEV attachment and infection, which suggests that TIM-1 mediates JEV infection in a PS-dependent way.

### 3.6. The Cytoplasmic Domain of TIM-1 Is Important for Enhancing JEV Entry

A recent study reported that TIM-1 mediates DENV endocytosis by ubiquitination at two lysines in its cytoplasmic tail [39]. To explore whether the cytoplasmic domain of TIM-1 functions in JEV infection, we generated a form of TIM-1 lacking the entire cytoplasmic tail (TIM-1 Δcyt). Our results demonstrate that, despite lacking its cytoplasmic domain, TIM-1 Δcyt is still distributed on the cell membrane (Figure 5A). However, the absence of the cytoplasmic tail inhibited the function of TIM-1 in mediating JEV entry (Figure 5F). The JEV virions entry into cells expressing TIM-1 Δcyt were not increased compared to control cells, and there was no colocalization of TIM-1 and JEV (Figure 5G). These results suggest that the cytoplasmic domain is required for JEV entry. Interestingly, we found that JEV replication in cells transiently expressing TIM-1 Δcyt was increased to 2.1-fold, and JEV production was increased approximately 3.2-fold and 4-fold at 24 h.p.i. and 36 h.p.i, respectively, compared to cells control. These data indicate that although the efficiency of TIM-1 Δcyt in mediating JEV infection was hindered, TIM-1 Δcyt can still enhance JEV infection (Figure 5B–D). Taken together, our data suggest that the cytoplasmic domain is important for JEV entry.

### 3.7. Knock-Down of TIM-1 in A549 Cells Impaired JEV Entry and Infection

**Figure 6:**
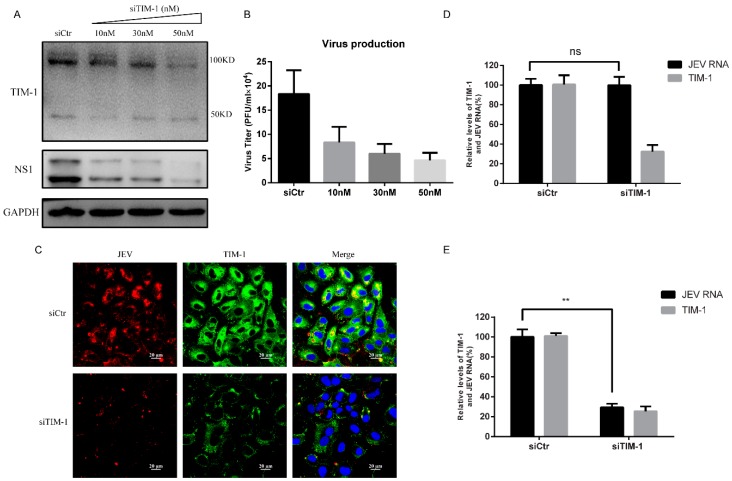
Knockdown of TIM-1 expression impairs JEV entry and infection. A549 cells were seeded in the 12-well plate, and they were transfected with TIM-1-specific siRNA of different concentrations or control siRNA at 50 nM using lipofectamine RNAiMAX Reagent. At 48 h post-transfection, cells were infected with JEV NJ2008 (MOI = 1) for 24 h. (**A**) Cell lysates were harvested to detect the level of JEV NS1 protein and cellular TIM-1 expression by Western blot analysis, using GAPDH as an internal control. One representative experiment out of three is shown; (**B**) production of progeny virions were determined by titering the supernatants of indicated cells in BHK-21 cells. Data are presented as mean ± SD from three independent experiments; (**C**) cells were transfected with TIM-1 siRNA or control siRNA for 48 h before being challenged with JEV NJ2008 (MOI of 10). At 24 h post-infection, cells were fixed and stained for E glycoprotein (red) and TIM-1 (green), followed by confocal microscopy analysis. Scale bars, 20 µm. A549 cells were transfected with TIM-1 siRNA or control siRNA for 48 h; (**D**) cells were incubated with JEV NJ2008 (MOI of 5) at 4 °C for 30 min and then washed with PBS three times; (**E**) cells were incubated with JEV NJ2008 with a MOI of 5 at 4 °C for 1 h and washed with PBS three times, then shifted to 37 °C for 15 min to allow JEV entry. Cells were treated with proteinase K (1 mg/mL) to remove non-internalized virions. Total RNA was extracted and used for quantification of JEV RNA, the efficiency of TIM-1 silencing and JEV attachment and entry were detected by qRT-PCR. Data are presented as mean ± SD from three independent experiments using t-test. ** *p* < 0.01.

Gain-of-function studies show that TIM-1 mediates JEV through ectopic expression in 293T cells. To further confirm that endogenous TIM-1 mediates JEV infection, we used TIM-1-specific small interfering RNA to silence endogenous TIM-1 expression in A549 cells and investigated the effect on JEV infection. We found that JEV infection was significantly inhibited at 24 h post-infection by silencing TIM-1 expression, as determined by decreased NS1 protein levels (Figure 6A). The level of JEV NS1 protein has a significant reduction compared to A549 cells transfected with nonspecific control (NSC) siRNA. Likewise, TIM-1 siRNAs resulted in reductions of JEV production in A549 cells, by approximately 55%, 67% and 74% at siRNA concentrations of 10 nM, 30 nM and 50 nM, respectively (Figure 6B). Using confocal microscopy, we also observed a marked reduction of JEV infection in A549 cells by silencing TIM-1 expression (Figure 6C). We then performed a JEV binding and internalization assay to examine whether endogenous TIM-1 functions during JEV binding and entry. At 48 h post-transfection, the level of TIM-1 mRNA was decreased by 74%, which suggested the TIM-1 silencing is successful. Interestingly, we detected no significant reduction of JEV attachment (Figure 6D), but knockdown of TIM-1 decreased the efficiency of JEV entry into A549 cells by 71% compared to A549 cells transfected with NSC siRNA (Figure 6E). Collectively, these data suggests that endogenous TIM-1 plays an important role in JEV infection, and there exist additional key receptors or cofactors for JEV binding in A549 cells. Moreover, the JEV internalization mediated by TIM-1 could be the major route of JEV entry into A549 cells.

## 4. Discussion

**Figure 7:**
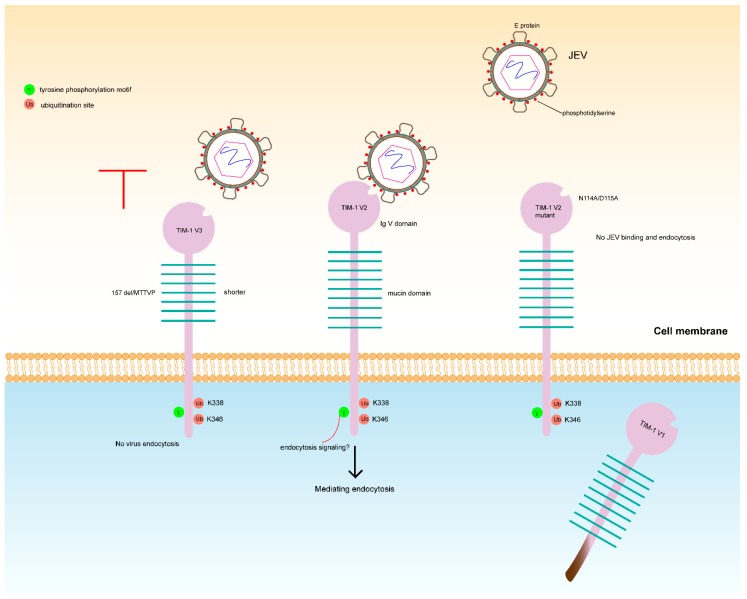
Hypothetical model of TIM-1 mediated JEV infection.

In the present study, we found that human TIM-1 plays a crucial role in JEV infection, and we proposed a hypothetical model of TIM-1-mediated JEV infection (Figure 7). Among the three TIM-1 variants cloned from A549 cells, TIM-1 V2 and V3 distribute on cell membrane and TIM-1 V2 significantly promotes JEV attachment, entry and infection. TIM-1 V3 slightly increases the efficiency of JEV attachment and infection. However, TIM-1 V1 distributes in the cytoplasm and cannot promote JEV infection. Additionally, we demonstrated that mutation of the TIM-1 V2 IgV domain with the Ptdser-binding pocket dampened JEV entry, which suggests that the process is Ptdser-dependent. Furthermore, the TIM-1 V2 cytoplasmic domain is also required for TIM-1 mediated JEV entry. Knock-down of endogenous TIM-1 expression by RNAi in A549 cells greatly impaired JEV entry and infection. Taken together, we show that TIM-1 promotes JEV infection as an entry cofactor.

Previous studies have suggested that polymorphisms of TIM-1 are associated with susceptibility to human immunodeficiency virus (HIV) and hepatitis A virus (HAV) infection. A 6-amino acid insertion/deletion in the mucin domain (157ins/dele MTTTVP) influenced the susceptibility of cells to virus. Also, a TIM-1 protein containing the 157insMTTTVP insertion bound to HAV more efficiently in binding assays and affected virus uncoating. Moreover, the 6-amino acid deletion provides protection against HIV-1 infection in clinical trials [21,32,33]. A 5-amino acid insertion (157ins MTTVP) in TIM-1 made cells more susceptible to viruses than a 6-amino acid 157ins MTTTVP [15]. In this study, we ectopically expressed the three TIM-1 variants in 293T cells, and only TIM-1 V2 (364aa) significantly promoted JEV binding, entry, and infection. There were almost no differences between the amino acid sequences of TIM-1 V2 and V1 except for a different cytoplasmic domain, and the N65D mutation did not impair virus entry [17]. Likewise, TIM-1 V3 had a 5-amino acid deletion (157dele MTTVP), a substitution of P174L and a mutation C52R. These differences ablated the ability of TIM-1 V1 and V3 to enhance JEV infection. Our study also shows that TIM-1 V1 was distributed in the cytoplasm of cells and not on the cell membrane, which may be due to the differences in the cytoplasmic domain. Thus, TIM-1 V1 does not function in mediating JEV infection. A stalk domain of sufficient length for the mucin domain is considered necessary for placing the IgV domain within the appropriate distance from cell surface, which allows interactions with viral particles [39]. Therefore, 157del MTTVP may impair the function of TIM-1 V3 in promoting JEV infection. It is also possible that the 5-amino acid deletion in the mucin domain influences the appropriate O-linked glycosylation of TIM-1 V3, which is crucial for proper protein folding and distribution on the cell membrane.

In the JEV binding and internalization assay, we observed that the ectopic expression of TIM-1 V2 in 293T cells can enhance the binding of JEV virions to host cells and, subsequently, their internalization. The key factors essential for TIM-1 proteins to function as a Ptdser receptor and mediate virus infection are their ability to bind Ptdser and attach to the cell surface [16,17,41]. Consistent with the crucial role of a Ptdser binding pocket in mediating virus entry, N114 and D115 were identified as important in Ptdser-dependent entry of several enveloped viruses by TIM-1 [16,19]. Our data show that TIM-1 N114A and D115A mutants hindered the function of TIM-1 in mediating JEV infection, and they aborted the ability of TIM-1 to mediate JEV internalization. Interestingly, TIM-1 mutants do not completely abort their ability to promote JEV infection during the late stages of infection. This indicates that after JEV internalization, TIM-1 may interact with certain cellular factors in the late stage of infection with other residues in the IgV domain but outside the MILIBS. Notably, we found that when TIM-1 lacks its entire cytoplasmic domain, it loses its ability to enhance JEV entry and reduce JEV infection. This suggests that the TIM-1 cytoplasmic domain plays a critical role in JEV entry and infection. Therefore, TIM-1 mediates JEV infection in a Ptdser-dependent manner, and the cytoplasmic domain of TIM-1 is also important for JEV entry.

Previous studies have shown that TIM-1 triggers *Flavivirus* uptake by binding to viral particles, and TIM-1 mediates *Flavivirus* endocytosis. It is constitutively internalized through clathrin-dependent endocytosis, which is a process that appears to rely on the phosphorylation of tyrosine residues within the cytoplasmic domain [42,43]. TIM-1 phosphorylation motifs are involved in the activation of T-lymphocytes through the PI3K/Akt pathway, which can prevent cell death caused by virus invasion. A recent study revealed that TIM-1 is ubiquitinated at two lysine residues within its cytoplasmic domain, and this modification is required for DENV endocytosis. The same study also demonstrated that TIM-1 interacts with STAM-1, which is a component of the ESCRT-0 complex involved in intracellular trafficking of ubiquitinated cargos and is required for DENV infection [35]. These previous studies indicate that TIM-1 may interact with other cellular factors after endocytosis. In the present study, our results are consistent with these previous findings. TIM-1 may be involved in intracellular trafficking following JEV endocytosis. JEV could initiate their life cycle earlier, which is similar to the mechanism utilized by TIM-1 for DENV or EBOV infection. *Flaviviruses* infect cells via receptor-mediated endocytosis and then are trafficked to endosomes. The acidic environment triggers conformational changes in the virus envelope E glycoprotein and induces the fusion of cells and the viral membrane [44,45]. Studies have shown that TIM-1 functions as an attachment factor in EBOV infection and that Niemann-Pick C1 (NPC1) acts as a fusion receptor. After internalization, TIM-1 can interact with NPC1 in intracellular vesicles, and it contributes to EBOV glycoprotein-mediated membrane fusion [46]. *Flavivirus* E glycoprotein is essential for viral particle attachment to target cells [47], even though interactions between cellular receptors and prM may be sufficient for entry in certain contexts [34]. Furthermore, TIM-1 can mediate Ebola virus infection by directly interacting with the viral glycoprotein GP through residues outside the MILIBS, which indicates that DENV and EBOV use distinct regions of TIM-1 [18,19]. Hence, TIM-1 may mediate virus infection in multiple mechanisms. In addition to the Ptdser-binding site, there are other regions in the IgV domain of TIM-1 that may interact with certain cellular factors. Relevantly, previous studies have suggested that the cytoplasmic domain of TIM-1 is required for DENV entry, but TIM-1 Δcyt is still able to enhance DENV infection [19,35]. We found that deletion of the cytoplasmic domain abolished the function of TIM-1 in promoting JEV entry. However, the efficiency of JEV infection is hindered to a lesser extent at 24 h.p.i. Taken together, we hypothesize that TIM-1 ubiquitination involves in JEV entry and is similarly to TIM-1-mediated DENV endocytosis. After JEV endocytosis, TIM-1 may interact with other cellular factors independent of the cytoplasmic domain, such as STAM-1, and this interaction may happen during JEV intracellular trafficking. This hypothesis could explain why TIM-1 V1 and V3 have the same IgV domain but do not function in mediating JEV infection.

In loss-of-function studies, we used RNAi technology to knock-down endogenous TIM-1 expression in A549 cells. Our results show that TIM-1 plays an important role in JEV entry and infection. These results confirm that TIM-1 promotes JEV infection as an entry cofactor. Our results also suggest that there exist other molecules serving as attachment factors in A549 cells. It is also postulated that JEV enter cells includes several steps, the first step is E glycoprotein-dependent (e.g., DC-SIGN), or PS-dependent (TIM-1 or other PS receptors) JEV attachment, which is then followed by the internalization mediated by primary receptors or entry cofactors (e.g., TIM-1). Recent studies have reported that TIM-1 is endogenously expressed in the epidermis primarily, if not exclusively, by keratinocytes of the basal layer [35]. This suggests that TIM-1 serves as an early stage JEV receptor in skin when a mosquito bites, which is important for the route of JEV transmission. Furthermore, TIM-1 is highly expressed in Th2 cells, and the interaction between JEV and TIM-1 may directly influence antigen presentation during the immune response. By extension, TIM-1 have also been shown to inhibit viral release, and the phosphatidylserine-binding capability of TIM-1 is essential for its inhibition of viral release [36]. TIM-1 promotes virus entry, and on the other hand, TIM-1 block virus replication and production. These studies indicate that TIM-1 may have dual functions in virus infection, and it is a complicated process. So how to regulate the dual function of TIM-1 or other Ptdser receptors in virus infection could be important for antiviral activities.

In conclusion, our findings highlight the potential importance of TIM-1 in the early stage of JEV infection, and TIM-1 promotes JEV infection by enhancing JEV attachment and entry into target cells. However, it is still unknown if TIM-1 induces intracellular signaling to favor subsequent virus infection and suppress innate immune responses. More studies are needed to elucidate the mechanism of TIM-1-mediated JEV infection, and the interactions between receptors and JEV need to be examined in vivo. Moreover, further understanding of the interactions between JEV and receptors may be important for the development of antiviral therapies.
